# Commercially available antiseptics show high *in vitro* efficacy against pathogens most commonly associated with canine and feline infectious keratitis

**DOI:** 10.3389/fvets.2025.1552230

**Published:** 2025-05-21

**Authors:** Hinrich Tönjes Wolff, Ana Cristina Piroth, Hilke Oltmanns, Jessica Meißner, Jutta Verspohl, Holger Andreas Volk, Claudia Busse

**Affiliations:** ^1^Small Animal Clinic, University of Veterinary Medicine Hannover, Foundation, Hannover, Germany; ^2^Department of Pharmacology, Toxicology and Pharmacy, University of Veterinary Medicine Hannover, Foundation, Hannover, Germany; ^3^Institute for Microbiology, University of Veterinary Medicine Hannover, Foundation, Hannover, Germany

**Keywords:** bacterial keratitis, antibiotic resistance, One Health, polyhexanide, povidone-iodine, N-acetylcysteine, hypochlorous acid

## Abstract

**Purpose:**

To determine the minimal bactericidal concentration (MBC) of polyhexanide (PHMB), povidone-iodine (PVP-I), N-acetylcysteine (NAC), and hypochlorous acid (HOCl) for bacterial species commonly found in canine and feline infectious keratitis.

**Methods:**

MBCs for clinical isolates of *Staphylococcus* (*S*.) *pseudintermedius* (*n* = 11), including 3 methicillin-resistant strains, *Pseudomonas* (*P*.) *aeruginosa* (*n* = 8), and *Streptococcus* (*Str*.) *canis* (*n* = 11), including the corresponding control strains, were examined. All testing substances were serially diluted in phosphate-buffered saline (PBS) and cation-adjusted Mueller–Hinton Broth (CAMHB) and inoculated with the bacterial suspension for 10 min. Afterwards, a neutralisation with Dey–Engley neutralising broth was performed, followed by plating onto Columbia sheep–blood agar. After incubation, plates were visually examined for bacterial growth. Tests were carried out in triplicate.

**Results:**

MBCs in PBS for polyhexanide ranged 0.8–1.6 mg/L for *S. pseudintermedius* and 1.6–3.2 mg/L for *P. aeruginosa* and *Str. canis*. For povidone-iodine, MBCs in PBS were observed at concentrations ranging 8–32 mg/L for *S. pseudintermedius* and *P. aeruginosa* and 8–16 mg/L for *Str. canis*. MBCs in PBS for NAC were recorded at a range of 6,400–12,800 mg/L for *S. pseudintermedius*, whereas those for *P. aeruginosa* and *Str. canis* ranged 3,200–6,400 mg/L. Results for HOCl in PBS ranged 0.4–1.6 mg/L for *S. pseudintermedius* and 0.4–0.8 mg/L for *P. aeruginosa* and *Str. canis*. MBCs in CAMHB for polyhexanide were found in the range between 3.2 and >12.8 mg/L, those for povidone-iodine between 6,400 and >12,800 mg/L, and for NAC between 6,400 and >12,800 mg/L, across the tested species. When dissolved in CAMHB, no antimicrobial effect could be observed for HOCl in concentrations up to 137.5 mg/L.

**Conclusion:**

All tested substances had an *in vitro* bactericidal effect against all three bacterial species with MBCs below known tolerated ocular concentrations when dissolved in PBS. Povidone-iodine and hypochlorous acid showed a marked reduction in their *in vitro* efficacy in the presence of protein. Nevertheless, our results provide a promising outlook on alternatives or adjuvants to antibiotics in ophthalmology that align with the One Health approach.

## 1 Introduction

Bacterial ulcerative keratitis is a commonly occurring and potentially vision- and globe-threatening disease (1, 2). Suter et al. (3) reported that nearly 20% of all ophthalmic small animals presented to their referral centre during a 20-year period exhibited corneal defects. In dogs and cats, bacterial keratitis is most commonly associated with *Staphylococcus* (*S*.) species, *Pseudomonas* (*P*.) species, and *Streptococcus* (*Str*.) species, although specific geographical and interspecies differences exist (3–13).

The mainstay of therapy for infectious ulcerative keratitis is topical antimicrobial therapy (14, 15). However, there are several factors that provide challenges to the treatment and prophylaxis of infectious keratitis. Due to the different modes of action and spectra of activity of available antibiotics, there is a lack of an antimicrobial with an effective range against all associated bacterial species (3–10). This is further complicated by frequently occurring co-infections (11). Some authors have therefore recommended the use of different topical antibiotics in conjunction with each other (16). Although interference between the different antibiotics has been reported and it may lead to a reduction in efficacy (17). Additionally, epitheliotoxicity is described for many topical antibiotics, and, therefore, these antibiotics will hinder corneal wound healing (18).

Antibiotic resistance is frequently reported for bacterial strains commonly seen in bacterial keratitis in dogs and cats (3–8, 10, 19). According to the World Health Organisation (WHO), antibiotic resistance is a major concern for human health (20). Considering the One Health approach, it is essential to combat antibiotic resistances in veterinary medicine (20), given the transfer of resistant bacterial strains between companion animals and humans (21–23). Therefore, the need for alternatives or adjuvant therapies to topical antibiotics arises.

Possible alternatives are antiseptics, for example, polyhexanide (PHMB), also known as polyhexamethylene biguanide, which is a synthetic polymer with a broad spectrum of antimicrobial activity against bacteria, some fungi, and protozoa (24, 25). Povidone-iodine (PVP-I) is still regarded as the predominant antiseptic for presurgical antisepsis in ophthalmology (26). N-acetylcysteine (NAC) is widely used in the treatment of corneal ulcerations due to its collagenase-inhibiting properties (27–30). Antimicrobial properties have also been reported in recent years (31–37). Hypochlorous acid (HOCl), a product of the respiratory burst of neutrophils (38–41), has an antimicrobial effect on a wide variety of bacteria, fungi, and viruses (38, 42–49).

## 2 Purpose

The purpose of this study was to determine the *in vitro* antimicrobial efficacy of the four antiseptics—polyhexanide, povidone-iodine, N-acetylcysteine, and hypochlorous acid—against bacterial species, most commonly associated with bacterial keratitis in dogs and cats. To investigate their potential as an alternative or adjuvant to topical antibiotics.

## 3 Materials and methods

### 3.1 Bacterial isolates

A total of 27 clinical isolates originating from infected canine (*n* = 26) and feline (*n* = 1) corneal ulcerations were evaluated together with the corresponding control strains. Investigated bacterial strains included *Staphylococcus* (*S*.) *pseudintermedius* (*n* = 11), *Streptococcus* (*Str*.) *canis* (*n* = 11), and *Pseudomonas* (*P*.) *aeruginosa* (*n* = 8), including the control strains (*S. pseudintermedius* DSM 25714, *Str. canis* DSM 20716, *P. aeruginosa* DSM 19880). Three isolates of *S. pseudintermedius* were methicillin and multidrug-resistant (MRSP). The clinical isolates were the same as those tested by Walter et al. (31), who reported MICs for NAC for these isolates. Isolates were stored at −70°C in glycerol and lysogeny broth at the Department of Pharmacology, Toxicology and Pharmacy of the University of Veterinary Medicine Hannover, Foundation, but were originally collected by a diagnostic laboratory (LABOKLIN, Bad Kissingen, Germany) (31).

### 3.2 Minimal inhibitory concentration (MIC)

Minimal inhibitory concentrations were tested using the microdilution procedure described in the Clinical and Laboratory Standards Institute (CLSI) protocol (50). In short: Stock solutions of the tested substances were prepared in sterile cation-adjusted Mueller–Hinton Broth (CAMHB; Mueller–Hinton–Bouillion, Carl Roth GmbH + Co. KG, Karlsruhe, Germany) and serially diluted for a total of eight concentrations per substance. Polyhexanide (Polihexanid-Lösung 20%; Fagron GmbH & Co. KG, Glinde, Germany) was tested at doubling concentrations from 0.1 to 12.8 mg/L. For povidone-iodine (PVP1-100G, poly(vinylpyrrolidone)-Iodine complex, Sigma–Aldrich Chemie GmbH, Steinheim, Germany), the pure substance was diluted to final concentrations from 100 to 12,800 mg/L. Hypochlorous acid was tested using a commercially available veterinary product (Vetericyn^®^VF plus eye & ear solution, Ecuphar GmbH, Greifswald, Germany) in concentrations up to 137.5 mg/L (0.01375%). It showed no observable antimicrobial effect in CAMHB. Further testing of the MIC values for this agent was therefore discontinued.

Each stock solution was freshly prepared on each day of testing.

For *Str. canis* inactivated chicken serum (chicken serum, sterile filtered, Bio&Sell GmbH, Feucht, Germany) was added to the stock solution (115 μL serum in 11.5 mL CAMHB) as described by Walter et al. in 2023 (31).

The bacteria were cultured on 5% Columbia sheep–blood agar (Columbia Agar with Sheep Blood, Oxoid Deutschland GmbH, Wesel, Germany) and incubated overnight at 37°C. A bacterial solution with a density of 0.5 McFarland units (MFU) was prepared in sterile saline (NaCl 0.9%, B. Braun Medical AG, Sempach, Switzerland) and 20 μL of this bacterial solution was added to 180 μL of the diluted stock solution in a sterile U-bottom 96-well cell culture plate with a lid (CELLSTAR, Greiner Bio-One GmbH, Frickenhausen, Germany). A negative control for each concentration was prepared by adding 20 μL of sterile saline instead of the bacterial solution. Eight positive controls were prepared by adding 20 μL of the bacterial solution to 180 μL of CAMHB. Afterwards, the 96-well plates were incubated at 37°C for 16–20 h and visually assessed for bacterial growth in the form of a button or turbidity formation. All tests were carried out in triplicate. The MIC is defined as the lowest concentration of an antimicrobial substance that inhibits visual growth of a microorganism (50); hence, the lowest concentration at which no turbidity or button formation could be detected was considered the MIC. Results for each bacterial isolate were recorded, and 96-well plates were documented photographically.

### 3.3 Validation of Dey–Engley neutralising broth

Prior to the MBC testing, a validation of the Dey–Engley neutralising broth (Dey-Engley-Neutralisierungs-Bouillon; Millipore, Merck KGaA, Darmstadt, Germany) was performed for double the highest tested concentration used in MBC testing of each substance (polyhexanide and HOCl at 25.6 mg/L, povidone-iodine and NAC at 25,600 mg/L) for all three control strains, following the ASTM International standard E 1054 – 2 (51).

### 3.4 Minimal bactericidal concentration (MBC)

For MBCs, stock solutions of polyhexanide, povidone-iodine, N-acetylcysteine, and hypochlorous acid were prepared and serially diluted in sterile phosphate-buffered saline (PBS) and in CAMHB, except for HOCl, which was only tested in PBS, as MIC testing had already shown a lack of efficacy for HOCl if dissolved in CAMHB.

All tests were carried out in triplicate. All 30 isolates were tested in PBS, whereas the MBCs in CAMHB were tested for all three control strains and all clinical isolates of *S. pseudintermedius* to validate the effect of CAMHB on the efficacy of the antiseptics.

Tested concentrations for polyhexanide ranged 0.1–12.8 mg/L. For povidone-iodine, two different concentration ranges were investigated depending on the solvent. Concentrations in PBS were 100-fold lower than those used for MIC testing, at 1–128 mg/L, whereas those in CAMHB were identical to the ones used in MIC testing, ranging 100–12,800 mg/L. NAC was tested in concentrations ranging 100–12,800 mg/L, regardless of whether dissolved in CAMHB or PBS. HOCl was tested solely in PBS in concentrations ranging 0.1–12.8 mg/L.

After bacterial cultivation as described above, bacterial solutions with a density of 0.5 MFU were prepared in PBS or sterile saline, depending on the solvent of the antiseptics, with sterile saline being used for testing in CAMHB. In a preliminary study, 0.5 MFU of the bacterial suspension of *S. pseudintermedius, P. aeruginosa*, and *Str. canis* were found to contain ~1.3 × 10^8^ CFU/mL, 8 × 10^7^ CFU/mL, and 3 × 10^7^ CFU/mL, respectively.

The bacterial suspension was diluted 100-fold. Afterwards, the testing solutions were inoculated in a one-to-one ratio with the bacterial solution. After 10 min of incubation at room temperature, 100 μL of the inoculated solution was neutralised by adding it to 900 μL of Dey–Engley neutralising broth. After at least 5 min of neutralisation, 100 μL were plated on Columbia sheep–blood agar to plate ~6,500 CFU/agar (6.5 × 10^4^ CFU/mL), 4,000 CFU/agar (4 × 10^4^ CFU/mL), or 1,500 CFU/agar (1.5 × 10^4^ CFU/mL), respectively, and incubated at 37°C overnight.

**Figure 1:**
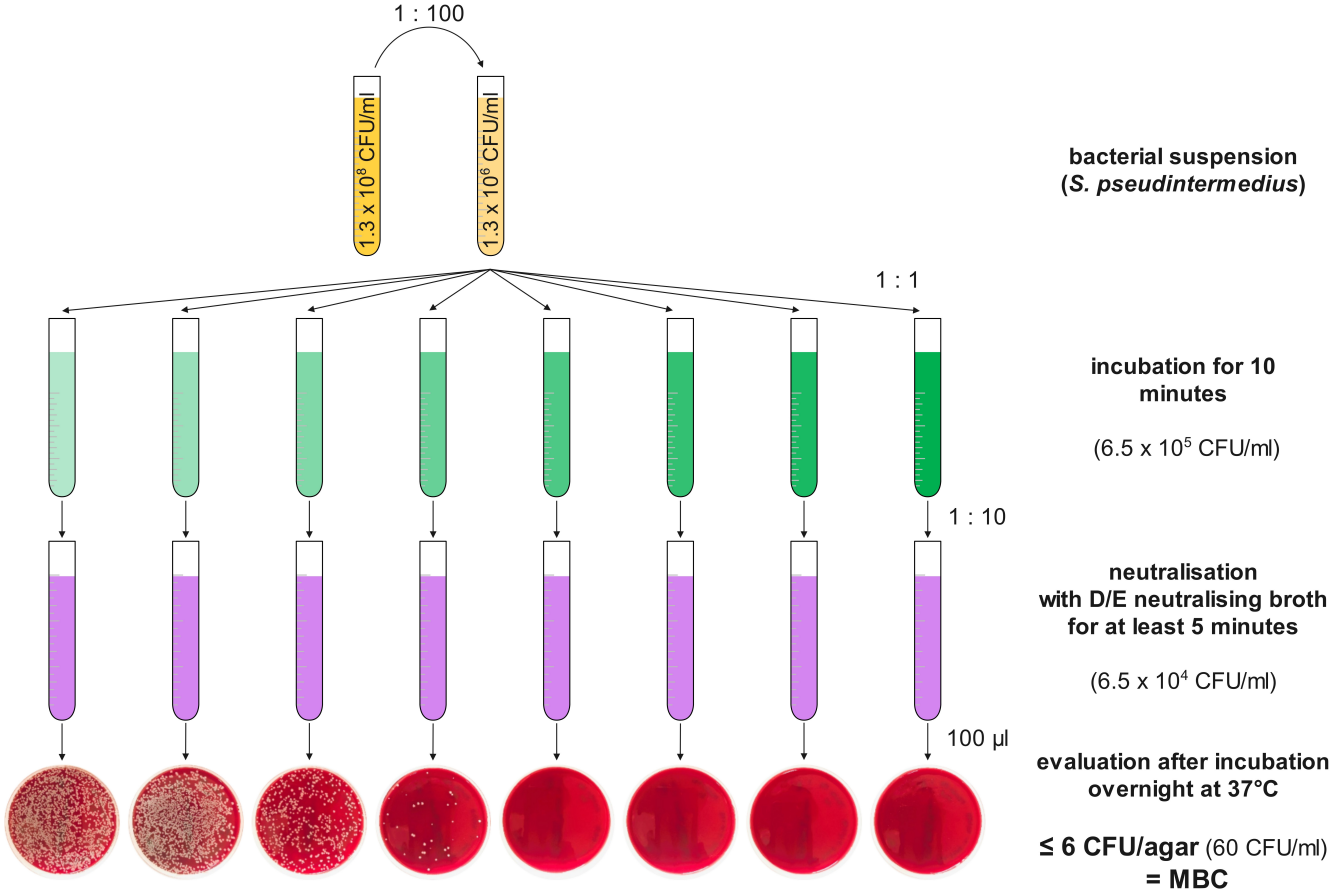
Schematic overview of the experimental design for testing MBCs (regardless of solvent) for *Staphylococcus pseudintermedius*. In yellow are the bacterial solutions starting with 0.5 MFU on the left, being 100-fold diluted to ~1.3 × 10^6^ CFU/mL. One of the antiseptics in rising (always doubling) concentrations from left to right is shown in green. The different concentrations of the antiseptics were inoculated in a one-to-one ratio with the bacterial solution. After 10 min of incubation, 100 μL of the antiseptic-bacterial mixture was neutralised with 900 μL of D/E-neutralising broth (in violet) for at least 5 min, before plating 100 μL on Columbia sheep–blood agar, incubation overnight, and visual evaluation of the CFUs/agar.

As MBCs are defined as a 3-log reduction (99.9%) of the viable bacterial load (52), evaluation was performed by counting and photographically documenting colonies on each agar and the MBCs threshold was determined to be equal to or < 6 CFU/agar (60 CFU/mL), 4 CFU/agar (40 CFU/mL), or 1 CFU/agar (10 CFU/mL) for *S. pseudintermedius, P. aeruginosa*, and *Str. canis*, respectively (see Figure 1).

### 3.5 Statistical analysis

The statistical analysis comprised a descriptive analysis of the collected data for MICs and MBCs (Excel 365, Version 2409, Microsoft 365 Apps for Enterprise), whereas the analysis for the validation of the neutraliser was performed according to ASTM International standard E 1054–2 (51) using SAS Studio software (version 3.8 on SAS 9.4 of the SAS System for Windows. Copyright©2012–2020, SAS Institute, Inc. SAS and all other SAS Institute, Inc. product or service names are registered trademarks or trademarks of SAS Institute, Inc., Cary, NC, USA).

## 4 Results

### 4.1 MICs

For polyhexanide, MICs for *S. pseudintermedius* ranged 1.6–3.2 mg/L, with all three MRSP strains having their MICs at 1.6 mg/L, whereas the MICs for *P. aeruginosa* and *Str. canis* ranged 6.4−12.8 mg/L and 3.2–12.8 mg/L, respectively. Additionally, polyhexanide was found to precipitate in concentrations equal to or higher than 25.6 mg/L in CAMHB in a preliminary experiment.

For povidone-iodine MICs for *S. pseudintermedius* and *Str. canis* were consistent at 6,400 mg/L, whereas those for *P. aeruginosa* ranged 3,200–12,800 mg/L.

CAMHB inactivated the antimicrobial effect of HOCl as no MIC or bactericidal effect could be observed in concentrations up to 137.5 mg/L for any of the tested isolates.

### 4.2 Validation of the Dey–Engley neutralising broth

Dey–Engley neutralising broth was confirmed to be an effective agent for neutralising polyhexanide, povidone-iodine, N-acetylcysteine, and hypochlorous acid within < 5 s, at least up to the tested concentrations, as there was no significant decrease in the bacterial load between the neutralised antiseptics and the positive control. The neutralisation broth had no intrinsic effect on bacterial growth and could therefore be classified as non-toxic to the tested bacterial species.

### 4.3 MBCs in CAMHB

MBCs in CAMHB for polyhexanide for *S. pseudintermedius* ranged 3.2–12.8 mg/L, for *P. aeruginosa* DSM 19880 at 6.4 mg/L, and for *Str. canis* DSM 20716 at 12.8 mg/L and above.

For povidone-iodine, MBCs for *S. pseudintermedius* ranged from 6,400 mg/L to >12,800 mg/L, whereas those for *P. aeruginosa* DSM 19880 were at 6,400 mg/L, and for *Str. canis* DSM 20716 at 12,800 mg/L and above.

MBCs of NAC for *S. pseudintermedius* were at 12,800 mg/L, with six of the 33 approaches being slightly above the threshold and therefore must be considered to be above 12,800 mg/L. The MBCs for *P. aeruginosa* DSM 19880 and *Str. canis* DSM 20716 were at 6,400 and 12,800 mg/L, respectively.

### 4.4 MBCs in PBS

MBCs in PBS for polyhexanide were at 0.8–1.6 mg/L for *S. pseudintermedius* and at 1.6–3.2 mg/L for *P. aeruginosa* and *Str. canis*.

For povidone-iodine MBCs in PBS were at 8–32 mg/L for *S. pseudintermedius* and *P. aeruginosa* and at 8–16 mg/L for *Str. canis*.

NAC was effective against *S. pseudintermedius* in concentrations ranging 6,400–12,800 mg/L when dissolved in PBS and against *P. aeruginosa* and *Str. canis* in concentrations ranging 3,200–6,400 mg/L. Additionally, it was found that the pH of 12,800 mg/L NAC in PBS was highly acidic at a pH of ~2.45, rising to pH 4.12 at 1,600 mg/L, where a significant increase to pH 6.48 occurred if further diluted to 800 mg/L.

**Figure 2:**
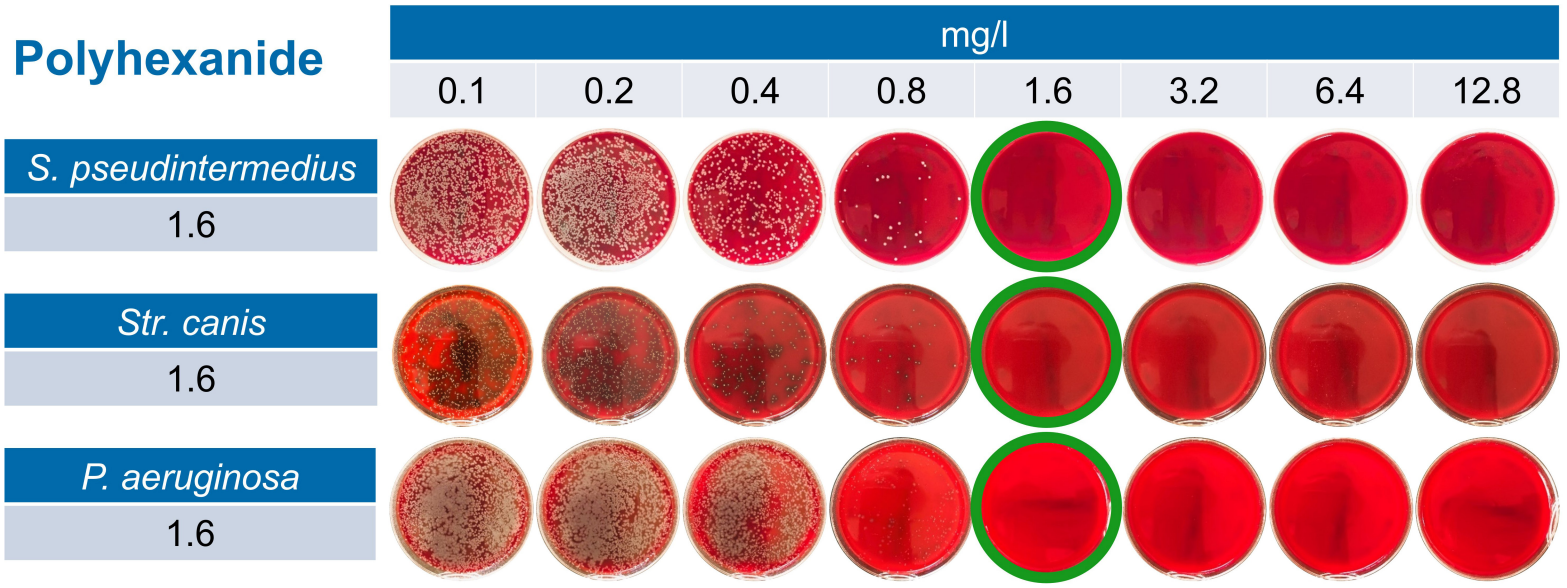
Exemplary results for polyhexanide in PBS across all three tested bacterial species. For *Staphylococcus pseudintermedius*, for example, one can observe a reduction in the CFU/agar starting at 0.4 mg/L of polyhexanide; nevertheless, a reduction by 99.9% (≤ 6 CFU/agar) is not observed until 1.6 mg/L. Therefore, 1.6 mg/L of polyhexanide dissolved in PBS is considered MBC for this isolate.

MBCs for hypochlorous acid for *S. pseudintermedius* were found at concentrations ranging 0.4–1.6 mg/L, with 1.6 mg/L being required for a total of four approaches of two isolates and for *P. aeruginosa* and *Str. canis* at 0.4–0.8 mg/L (see also Figure 2 and Table 1).

**Table 1:** Results for MICs and MBCs in CAMHB and PBS in mg/L.

		***Staphylococcus pseudintermedius* (n = 11)**	***Pseudomonas aeruginosa* (n = 8)**	***Streptococcus canis* (n = 11)**
Polyhexanide	MIC	1.6–3.2 {3.2} [3.2]	6.4–12.8 {6.4} [6.4]	3.2–12.8 {6.4} [6.4]
	MBC in CAMHB	3.2–12.8 {6.4} [6.4]	{6.4}^*^	{Between 12.8 and >12.8}^*^
	MBC in PBS	0.8–1.6 {1.6} [1.6]	1.6–3.2 {3.2} [1.6]	1.6–3.2 {1.6} [1.6]
Povidone-iodine	MIC	6,400 {6,400} [6,400]	3,200–12,800 {6,400} [6,400]	6,400 {6,400} [6,400]
	MBC in CAMHB	Between 6,400 and >12,800 {>12,800} [>12,800]	{6,400}^*^	{12,800–12,800}^*^
	MBC in PBS	8–32 {32} [16]	8–32 {32} [16]	8–16 {8} [8]
NAC	MBC in CAMHB	Between 12,800 and > 12,800 {Between 12,800 and >12,800} [12,800]	{6,400}^*^	{12,800}^*^
	MBC in PBS	6,400–12,800 {6,400} [6,400]	3,200–6,400 {6,400} [3,200]	3,200–6,400 {6,400} [6,400]
HOCl	MIC	Not obtainable	Not obtainable	Not obtainable
	MBC in CAMHB	Not obtainable	Not obtainable	Not obtainable
	MBC in PBS	0.4–1.6 {0.8} [0.8]	0.4–0.8 {0.8} [0.4]	0.4–0.8 {0.8} [0.4]

In square brackets, modal values if applicable. In curly brackets, the values of the respective reference strain. *Only tested in triplicate for the corresponding control strain (n = 1), hence modal values are not applicable. In grey, values are wholly or partially above the highest tested concentration. MIC, minimal inhibitory concentration; MBC, minimal bactericidal concentration; PBS, phosphate buffered saline; CAMHB, cation-adjusted Mueller Hinton Broth; NAC, N-acetylcysteine; HOCl, hypochlorous acid.

## 5 Discussion

Our results show that polyhexanide, povidone-iodine, N-acetylcysteine, and hypochlorous acid have a strong *in vitro* antimicrobial effect against all tested bacterial species, most commonly associated with bacterial keratitis in dogs and cats, especially when tested in PBS. Notably, all tested methicillin-resistant strains of *S. pseudintermedius* were equally susceptible to all tested antiseptics. All four antiseptics—polyhexanide (53–55), povidone-iodine (33–37), NAC (38, 47, 56, 57), and HOCl (38, 47, 56, 57)—are reported to be effective against biofilms in various locations and settings, which is of particular interest as many of the bacterial species associated with infectious keratitis are known to form biofilms (58–61).

In this study, MBCs were defined as a 3 log reduction of the viable bacterial load as stated by the Clinical and Laboratory Standards Institute (CLSI) (52). Although other definitions of MBCs exist, for example, in DIN EN 1040:2005 (62), we chose the definition provided by the CLSI as it is recognised as a main standard for antimicrobial testing (63, 64) and to improve potential inter-study comparability.

### 5.1 Polyhexanide (PHMB)

Polyhexanide (PHMB) is used as an antiseptic and disinfectant in a wide array of medical and non-medical settings (24, 25, 65, 66).

It interacts with negatively charged bacterial membranes, leading to their disruption (67, 68), as well as translocating across the membrane and interacting with the genetic material (66, 69, 70), while having a relatively low activity on mammalian cell membranes (71, 72).

In human ophthalmology, it is used as the mainstay for the treatment of Acanthamoeba keratitis (67, 68) and as an alternative for presurgical antisepsis (66, 69, 70), where it is reported to have an extended duration of antisepsis on the ocular surface compared to povidone-iodine (69). Despite being used for years, no bacterial resistance has been reported until now (24) and there is no evidence of resistance development after repeated incubation with polyhexanide (73). One study has shown a protective effect of polyhexanide on human keratocytes being co-cultured with *Staphylococcus aureus* (74).

Polyhexanide was deemed to be safe for use on the ocular surface in concentrations up to 0.08% (800 mg/L) in human trials (71) and no cytotoxicity to the corneal epithelium was found in an *in vitro* and *ex vivo* study for 0.04% (400 mg/L) after an exposure time of 30 min (72), therefore giving a significant margin over our observed MICs and MBCs regardless of solvent.

Polyhexanide is not readily biodegradable, but is still considered to have a low potential for bioaccumulation (75). However, it is considered very toxic to aquatic life with long-lasting effects (75). Although the environmental risk of eyedrops might be less significant, care should be taken when disposing of leftovers.

### 5.2 Povidone-iodine (PVP-I)

Povidone-iodine (PVP-I) exerts its antimicrobial effect against bacteria, fungi, protozoa, and some viruses (76–79) by gradually releasing free iodine (77, 80), which rapidly penetrates the microorganisms and attacks key groups of proteins, nucleotides, and fatty acids, leading to cell death (77, 81). Despite being used for decades, no induced resistance development has been reported (73, 77, 82).

It is still regarded as the predominant antiseptic for presurgical antisepsis in both veterinary and human ophthalmology (26, 82–84). Additionally, it is also used in the treatment of human ophthalmia neonatorum, some cases of keratitis, and conjunctivitis (26).

Concentrations used and considered to be safe on the ocular surface are somewhat inconsistent. Still, the American Academy of Ophthalmology and European Society of Refractive Surgeons guidelines recommend a concentration of 5% (50,000 mg/L) for corneal antisepsis (83, 84). Foja et al. (72) did not find evidence that using 1 and 5% povidone-iodine for up to 2 min had a cytotoxic effect on porcine corneas, whereas 5% povidone-iodine caused damage to the ocular surface of rabbits in a time-dependent manner (for 3 and 10 min) in another study (85). Our MBCs in PBS are therefore ~1,500-fold lower than the currently recommended concentrations in human ophthalmology. In contrast, the MBCs measured if dissolved in CAMHB are closer to the maximum tolerated concentrations. This is most likely due to protein interference, as discussed later.

As iodine occurs naturally in the environment in relatively high concentrations, the actual risks to the environment arising from the use of iodine are considered acceptable according to the European Chemicals Agency (ECHA). Nevertheless, iodine is considered toxic to aquatic life with long-lasting effects (86) and therefore should not be released carelessly into the environment.

### 5.3 N-acetylcysteine (NAC)

N-Acetylcysteine (NAC) is an acetylated form of the amino acid L-cysteine and an active precursor of glutathione, which acts as an antioxidant (29, 31, 87, 88). NAC is used regularly in medicine for its mucolytic, antioxidative, and chelating properties (31, 87–92) and additionally has anti-inflammatory properties by the mediation of cytokine release (31, 87, 89, 93).

In ophthalmology, it is widely used in the treatment of corneal ulcerations due to its collagenase-inhibiting properties (27–30), which makes it a mainstay for the treatment of melting ulcers (94). It is also used in the context of dry eye disease and meibomian gland dysfunction (29). One study reported a significant acceleration of corneal wound healing in dogs (95). In recent years, several studies have described antimicrobial properties of NAC against a wide array of bacterial species, including species commonly associated with infectious keratitis in dogs and cats (31–37).

In our study, we could confirm a bactericidal effect of NAC, in concentrations of 3,200–12,800 mg/L (0.32–1.28%) when dissolved in PBS. pH values of the effective concentrations of NAC in PBS ranged from approximately pH 2.45–3.12. The significance of this finding is unclear, and further research is required to assess the effect of the pH values on the bacterial isolates and the efficiency of the NAC itself.

NAC is safe for use on the ocular surface in concentrations of at least 2.5% (96) (25,000 mg/L) and possibly even up to concentrations of 20%, although there are conflicting reports (95, 97). Our reported MBCs are lower than concentrations currently used in veterinary ophthalmology for their collagenase-inhibiting properties. Due to the low margin over the MBCs, NAC is more likely to be used as a prophylactic agent in corneal ulcers, to prevent secondary infection, rather than as a sole treatment option for infectious keratitis.

### 5.4 Hypochlorous acid (HOCl)

Hypochlorous acid (HOCl) belongs to the group of reactive species (40, 41). It is used as an antiseptic and disinfectant in many medical and non-medical applications (38, 56, 57, 98–101). Additionally, HOCl has been shown to have anti-inflammatory properties (45), as well as favourable effects on fibroblast and keratinocyte migration (38).

In human ophthalmology, it is currently mainly used in the treatment of blepharitis (49, 102–104) and fungal keratitis (44–46). In our study, HOCl was highly effective against all three tested bacterial species in concentrations as low as 0.4–1.6 mg/L when dissolved in PBS. However, when dissolved in CAMHB, no antibacterial effect could be observed in concentrations up to 137.5 mg/L. This effect is most likely due to the interference of organic matter and protein (105), as discussed later. No concerns have been raised about the ocular toxicity of HOCl (106). Wang et al. (39) found HOCl to be non-irritating and non-sensitising to the ocular surface in various animal models in all tested concentrations up to 0.013% (130 mg/L).

Production of hypochlorous acid is relatively inexpensive, though the solution needs to be stabilised (107) and degrades rapidly when coming into contact with organic matter, therefore, the ECHA assesses the potential environmental risk as acceptable (108, 109). Hence, it is a great candidate for rinsing eyes affected by corneal ulcers, as it first lowers the protein content of the tear film and exerts antimicrobial effects afterwards, therefore leading to a reduction of the bacterial load by two different pathways. It has also been reported to reduce the bacterial load without affecting the bacterial diversity (49) or the biodiversity in the meibomian gland secretions of patients with internal hordeolum (110), making it also interesting for preventing secondary infection on the ocular surface.

### 5.5 Protein interference

For all four antiseptics, differences between the MICs and MBCs in CAMHB and those in PBS were observed. Notably, for HOCl, no antimicrobial effect could be observed up to concentrations of 137.5 mg/L when dissolved in CAMHB, compared to a maximum observed MBC of 1.6 mg/L in PBS. For povidone-iodine, over 100-fold higher concentrations were required to achieve MBCs in CAMHB compared to PBS. This trend was also observable for polyhexanide and NAC, although differences were less marked.

This discrepancy in the effectiveness of the antiseptics depending on the solvent might be due to the protein content of ~2% in CAMHB compared to the protein-free environment of PBS. Since interference between proteins and the antiseptics might lead to a reduction in their efficacy (105, 111).

The protein content of the healthy canine eye ranges from 2.8 to 4.03 μg/μL (112) (0.28–0.403%) and is, therefore, approximately five times lower than the protein content of CAMHB. Hence, a reduction in the efficiency of the antiseptics, especially povidone-iodine and HOCl, is expected upon contact with the tear film. Nevertheless, the reported MBCs in CAMHB for polyhexanide are well, and for povidone-iodine and NAC, they were still below the documented highest tolerated respective concentrations on the ocular surface.

### 5.6 *In vivo* effects

In addition to possible interference with organic matter, one must consider the further dilution of the antiseptic upon application onto the ocular surface due to the tear film. In commercially available eyedroppers, the volume of one drop ranges from 26.4 μL up to 69.4 μL (113), with another study finding an average drop volume of 39.0 μL (114). The reported median volume of the tear film of dogs and cats is 65.3 and 32.1 μL, respectively (115), while the reported volumetric capacity of the canine palpebral fissure was 31.3 ± 8.9 μL (15–45 μL) in healthy beagles (116). Therefore, the further dilution of the antiseptics is estimated to be approximately at a one-to-one to one-to-two ratio. As our reported MBCs (especially in PBS) are significantly lower than the reported concentrations deemed to be safe on the ocular surface, there should be a sufficient margin to accommodate the dilution by the tear film.

A further aspect, which needs to be taken into account, is the limited contact time achievable *in vivo* due to the reflex tear film turnover time, which has been reported to be ~50.0%/min in dogs and cats (115). Therefore, further research is required to determine the time-kill kinetics of the antiseptics on the bacterial species associated with canine and feline infectious keratitis.

Furthermore, it is unclear what effect the antiseptics might have on the tear film quality and composition, as well as on the microbiome in the canine and feline eye. Therefore, further research should be performed in this regard. Additionally, other *in vivo* aspects, for example, the lipid component of the tear film, might affect the antiseptics; further research is required in this regard.

### 5.7 Study limitations

Limitations of this study are its *in vitro* nature, as the *in vivo* effects and required concentrations might differ, the relatively small sample size, and most of the clinical isolates being of canine origin. Although just one feline isolate was tested, its MIC and MBC were found to be similar to those of the canine isolates and the respective reference strain. Additionally, since MICs and MBCs for the reference strains for all tested bacterial strains were similar to the MICs and MBCs of the clinical isolates, and interspecies variability toward the bactericidal effect was low, we consider it likely for other clinical isolates of feline origin of the tested bacterial strains to behave likewise. Further research toward this aspect is required.

## 6 Conclusion

Our results show a potent *in vitro* antimicrobial effect of polyhexanide, povidone-iodine, N-acetylcysteine, and hypochlorous acid when dissolved in PBS against *S. pseudintermedius*, including methicillin-resistant strains, *P. aeruginosa*, and *Str. canis*, which are all commonly associated with canine and feline infectious keratitis. The recorded MBCs were well below known tolerated ocular concentrations. Therefore, the tested antiseptics might be an ideal alternative or addition to topical antibiotics in the treatment and prophylaxis of infectious keratitis, especially as many of the studied substances have been reported to have additional beneficial effects on corneal healing, inflammation, prevention of melting of corneal ulcers, and more. However, some antiseptics, notably povidone-iodine and hypochlorous acid, show a marked reduction in their *in vitro* efficacy when dissolved in a protein-containing broth, which might indicate a lower efficacy after contact with the tear film *in vivo*. Further research is required to assess several *in vivo* factors and time-kill kinetics. All in all, antiseptics might play an essential role in reducing the use of topical antibiotics in veterinary ophthalmology, therefore combating antimicrobial resistance and its development in line with the One Health approach.

## Data Availability

The raw data supporting the conclusions of this article will be made available by the authors, without undue reservation.
